# Supplementary material for: Targeting ADAR stability: a novel approach to combat glioblastoma
Source: Front Oncol. 2026 Aug 6;16:1827793. doi: 10.3389/fonc.2026.1827793 (PMC13489870; doi:10.3389/fonc.2026.1827793)
Supplement: Supplementary file 1 [file SupplementaryFile1.zip › Supplementary file 1/Pentose_and_glucuronate_interconversions.pdf]

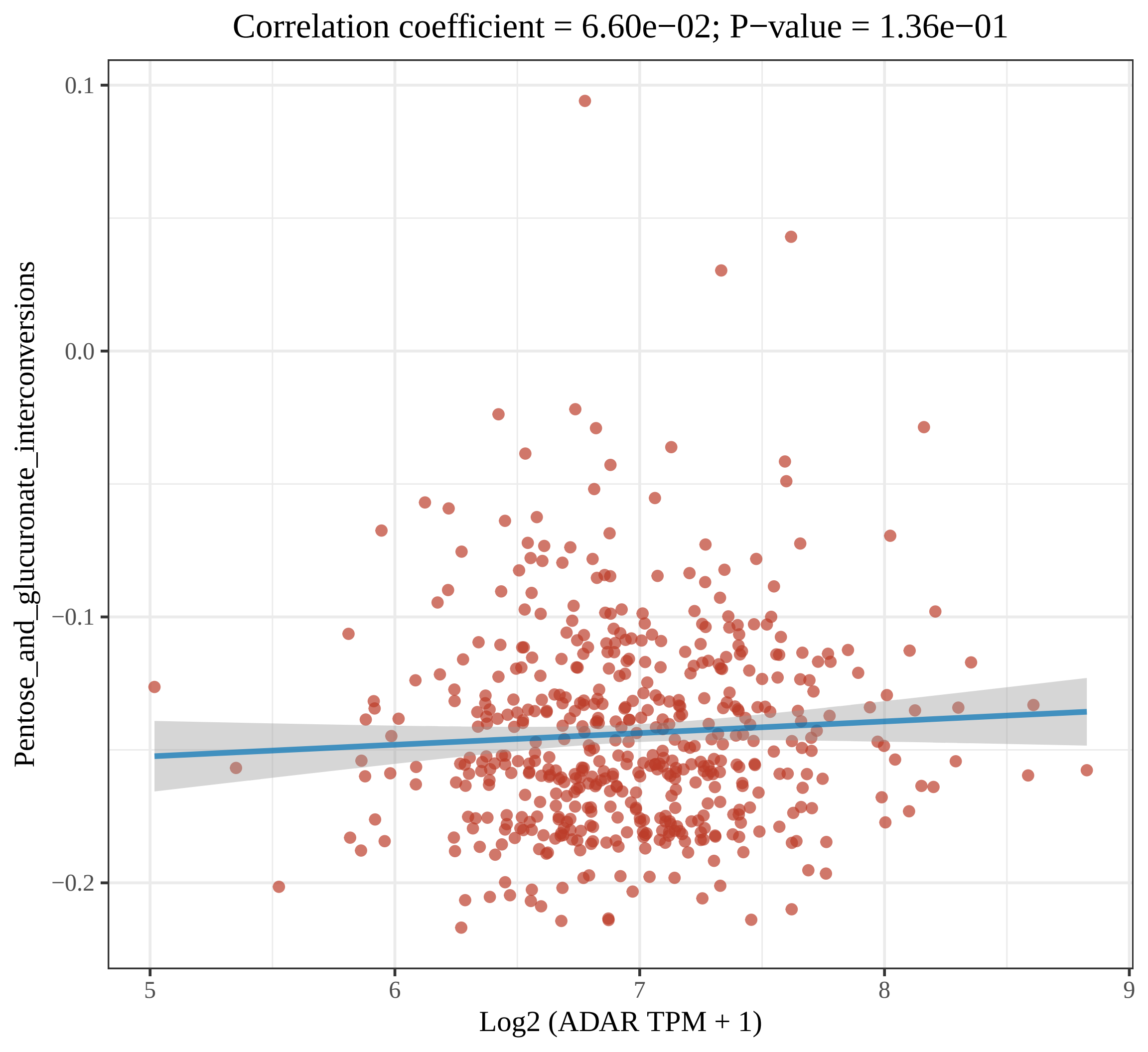

Correlation coefficient = 6.60e−02; P−value = 1.36e−01
0.1
0.0
−0.1
−0.2
Pentose_and_glucuronate_interconversions
5
6
7
8
9
Log2 (ADAR TPM + 1)
